# Exploring the Ecological Implications of Microbiota Diversity in Birds: Natural Barriers Against Avian Malaria

**DOI:** 10.3389/fimmu.2022.807682

**Published:** 2022-02-17

**Authors:** Vaidas Palinauskas, Lourdes Mateos-Hernandez, Alejandra Wu-Chuang, José de la Fuente, Justė Aželytė, Dasiel Obregon, Alejandro Cabezas-Cruz

**Affiliations:** ^1^ Nature Research Centre, Akademijos 2, Vilnius, Lithuania; ^2^ ANSES, INRAE, Ecole Nationale Vétérinaire d’Alfort, UMR BIPAR, Laboratoire de Santé Animale, Maisons-Alfort, France; ^3^ SaBio, Instituto de Investigación en Recursos Cinegéticos IREC-CSIC-UCLM-JCCM, Ciudad Real, Spain; ^4^ Department of Veterinary Pathobiology, Center for Veterinary Health Sciences, Oklahoma State University, Stillwater, OK, United States; ^5^ School of Environmental Sciences, University of Guelph, Guelph, ON, Canada

**Keywords:** anti-α-Gal antibodies, avian malaria, gut microbiota, transgenerational immunity, protective immunity

## Abstract

Natural antibodies (Abs), produced in response to bacterial gut microbiota, drive resistance to infection in vertebrates. In natural systems, gut microbiota diversity is expected to shape the spectrum of natural Abs and resistance to parasites. This hypothesis has not been empirically tested. In this ‘Hypothesis and Theory’ paper, we propose that enteric microbiota diversity shapes the immune response to the carbohydrate α-Gal and resistance to avian malaria. We further propose that anti-α-Gal Abs are transmitted from mother to eggs for early malaria protection in chicks. Microbiota modulation by anti-α-Gal Abs is also proposed as a mechanism favoring the early colonization of bacterial taxa with α1,3-galactosyltransferase (α1,3GT) activity in the bird gut. Our preliminary data shows that bacterial α1,3GT genes are widely distributed in the gut microbiome of wild and domestic birds. We also showed that experimental infection with the avian malaria parasite *P. relictum* induces anti-α-Gal Abs in bird sera. The bird-malaria-microbiota system allows combining field studies with infection and transmission experiments in laboratory animals to test the association between microbiota composition, anti-α-Gal Abs, and malaria infection in natural populations of wild birds. Understanding how the gut microbiome influences resistance to malaria can bring insights on how these mechanisms influence the prevalence of malaria parasites in juvenile birds and shape the host population dynamics.

## Introduction

Understanding the mechanisms involved in host-microbiota interactions is important as beneficial microbes can influence the health, fitness, and immunity of vertebrate hosts (1), and although less known, host microbiota can also facilitate pathogen infection (2). One important contribution of microbiota to immunity is the induction of natural antibodies (Abs). These Abs can target glycans on the surface of pathogens, protecting the host from infection. For example, the presence of Gram-negative bacteria expressing the disaccharide Galα1-3Gal (α-Gal) in gut microbiota of animals that do not produce endogenous α-Gal induces the production of natural Abs specific to the glycan (3). Humans, fish and birds lack the enzymatic machinery necessary for α-Gal synthesis (3), which allows for the production of anti-α-Gal Abs (4–8). The broad distribution of bacterial α-1,3-galactosyltransferase (α1,3GT) genes in the human gut microbiome (9), and the high levels of circulating immunoglobulin (Ig) M and IgG directed against the glycan in healthy adults further suggest a link between microbiota and humoral immunity (10).

High levels of natural anti-α-Gal IgM are associated with protection from *Plasmodium falciparum* infection in humans (11, 12). In addition to mosquito-borne *Plasmodium* spp (11)., other vector-borne pathogens such as *Borrelia* spp (13)., transmitted by ticks; *Leishmania* spp (14)., transmitted by sandflies; and *Trypanosoma* spp (15–17)., transmitted by triatomes; express α-Gal on their surface. Induction of high levels of anti-α-Gal Abs by α-Gal immunization protects against experimental infection by these pathogens in mice (8). Oral administration of bacteria expressing high levels of α-Gal recapitulates the etiology of natural anti-α-Gal Ab production in α1,3GT-deficient mice (do not produce endogenous α-Gal) (11), zebrafish (18) and turkeys (7). This microbiota manipulation also induces protective immunity, as gut colonization of α1,3GT-deficient mice by *Escherichia coli* O86:B7 elicited a protective anti-α-Gal IgM response that significantly reduced malaria transmission (11). Furthermore, anti-α-Gal IgM triggered complement-mediated lysis of *Plasmodium* sporozoites associated with sterile protection against murine malaria (11).

The ecological impact of gut microbiota diversity and its association with anti-α-Gal immunity remains to be tested. Birds represent over 30% of known tetrapod diversity with 10 425 described species and more than 20 000 subspecies varieties (19). Their different migratory behaviors, habitats and diets influence microbiota composition diversity (20, 21), which in turn may have large impact on resistance to parasites (22). However, the mechanisms driving bird-parasite-microbiota interactions remain poorly characterized. There are more than 50 avian malaria species and new species are discovered every year (23). Moreover, based on mitochondrial genome analysis of avian *Plasmodium* spp., there might be many more species than previously thought (24). Field and experimental studies reveal that the host specificity of these pathogens varies from strict specialists infecting a single bird species to generalists infecting more than 300 distantly-related bird species (23, 24). Notably, avian malaria infections are common in some bird species, but not in others (25), and the causative factors driving these differences are not clear. In this ‘Hypothesis and Theory’ paper, we propose to use the bird-malaria-microbiota system to dissect the ecological implications of gut microbiota diversity to anti-α-Gal response, resistance to *Plasmodium* infection and the inter-generational effect of such microbiota-mediated immunity. Within the text, “microbiome” refers to the microorganisms and their metagenome (i.e., when the genes are known and/or are being referred to intentionally) whereas “microbiota” refers only to the microbes themselves (i.e., when the genes are unknown and/or there is no intention to refer to all or any of them in particular).

## The Puzzling Origin of the Glycan α-Gal in *Plasmodium* spp.

Enzymatic glycosylation of proteins and lipids is a common and important biological process in prokaryotic and eukaryotic organisms (26). In general, the identification of genes encoding for enzymes with α1,3GT activity is challenging for several reasons. First, prokaryotic and eukaryotic α1,3GT genes and encoded proteins share little structural homology (26–30). Second, within defined taxonomic groups (i.e., prokaryotic), the α1,3GT diversity is very high with some cases in which single bacterial species have several α1,3GT enzymes translated from different, non-related, genes (9). Third, α1,3GT from different eukaryotic lineages do not share immediate common ancestors. For example, α1,3GT encoded by the gene *ggta1* is responsible for the production of the α-Gal epitope in non-primate mammals, Lemurs and New World monkeys (10). However, no genetic trace of *ggta1* could be found in the genomes of fungi (29), arthropods (e.g., ticks (27) and mosquitoes (31) or Apicomplexan (e.g., *Plasmodium*) (31) organisms expressing α-Gal. This suggests that the capacity for α-Gal synthesis have evolved independently, and multiple times, during prokaryotic and eukaryotic evolution.

Empirical research using *Plasmodium berghei* ANKA and α1,3GT-deficient mice revealed that anti-α-Gal Abs target *Plasmodium* sporozoites for complement-mediated cytotoxicity in the skin after transmission by *Anopheles* mosquitoes (11). This complement-mediated immune reaction prevented sporozoites from reaching host hepatocytes and complete the next step of the parasitic life cycle in the liver. Specific binding of anti-α-Gal Abs requires the presence of α-Gal on the surface of *Plasmodium* sporozoites. However, the origin of *Plasmodium* α-Gal has not been completely elucidated. Particularly, it is not yet clear whether the glycan α-Gal is: (*i*) directly synthesized by the parasite, or (*ii*) synthesized by the vector and metabolically incorporated as a terminal group in glycoproteins and/or glycolipids of the parasite. In the study by Yilmaz et al. (11), the salivary glands of *Plasmodium*-infected *Anopheles* mosquitoes contained α-Gal, but low levels of this glycan were detected also in the salivary glands of uninfected mosquitoes. Furthermore, α-Gal was identified on the surface of sporozoites of the human pathogen *P. falciparum* 3D7, and of the rodent pathogens *P. berghei* ANKA and *Plasmodium yoelii* 17XNL (11). This raised the possibility that both, *Plasmodium* parasites and mosquito vectors, have the enzymatic machinery for endogenous α-Gal synthesis.

A recent study based on protein similarity analysis indicated the presence of homologous to three *Ixodes scapularis* proteins with α1,3GT activity in two mosquito vectors, *Aedes aegypti* and *Anopheles gambiae* (31). However, the genus *Plasmodium* lacked proteins homologous to the three *I. scapularis* proteins with α1,3GT activity (31). Empirical evidences support that the genes identified in *I. scapularis*, *b4galt7*, *a4galt-1* and *a4galt-2*, have direct α-Gal-synthetizing activity and/or participate in the α-Gal synthesis pathway in ticks (27). First, heterologous expression of these genes in bacterial and human α-Gal-negative cells induced *de novo* synthesis of α-Gal. Second, transcriptional upregulation of the three genes in fed ticks was associated with increased α-Gal levels in tick tissues. Third, RNA interference-mediated silencing of the three genes was associated with reduction of α-Gal levels in tick tissues. Fourth, simultaneous silencing of the three genes reduced the α-Gal levels in the tick cell line IRE/CTVM20 (27). It is important to mention that the α1,3GT activity of mosquito homologous have not been experimentally tested. However, recent studies further suggest that *Plasmodium* and mosquitoes have both the capacity to produce α-Gal epitopes (32). Disruption of α-Gal production in mosquitoes was not associated with significant reduction of α-Gal levels in *Plasmodium* (32).

**Figure 1:**
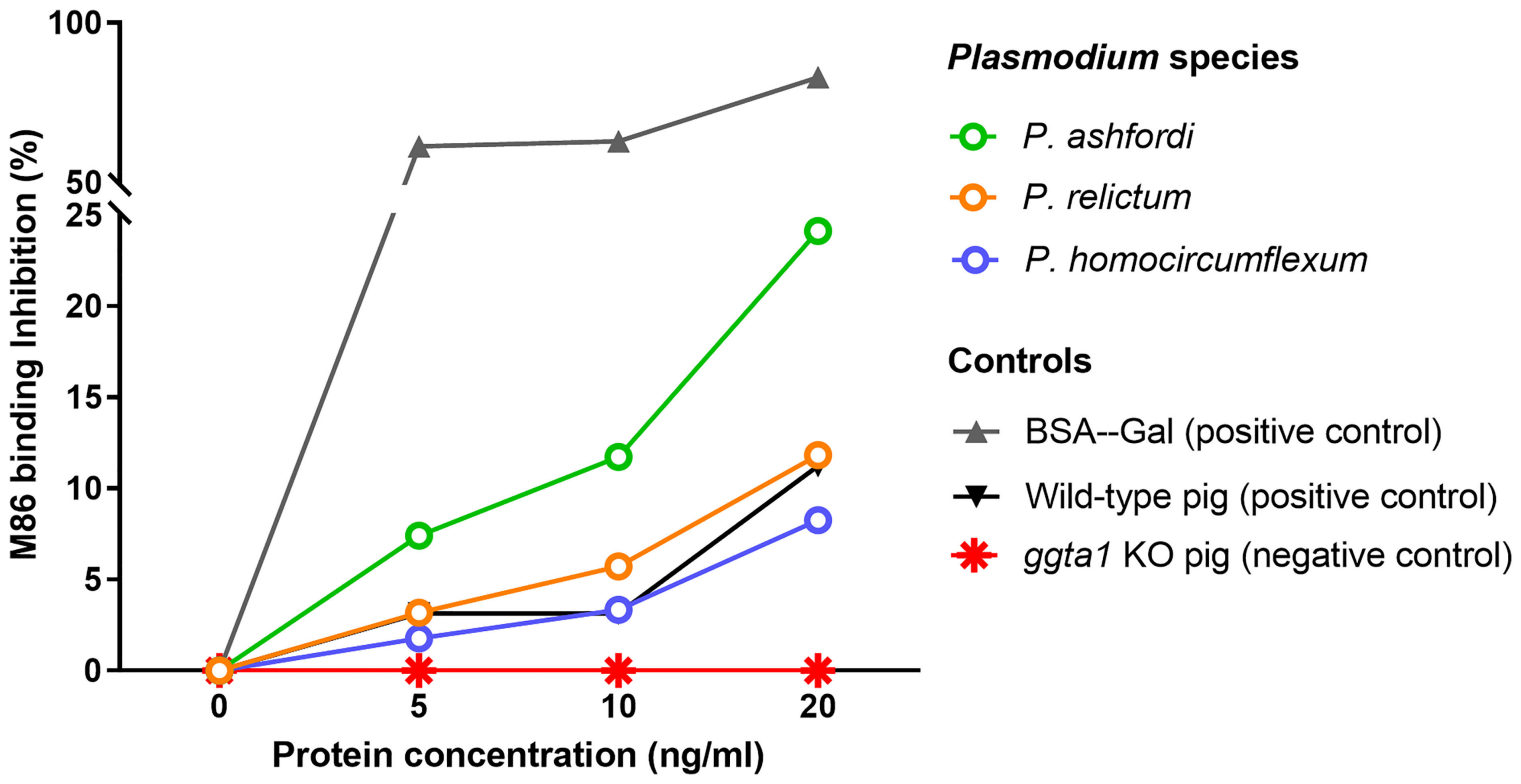
Presence of α-Gal in avian *Plasmodium* species. The levels of α-Gal in protein extracts from different *Plasmodium* species, *P. ashfordi* (GRW2), *P. relictum* (SGS1) and *P. homocircumflexum* (COLL4), are presented. The inhibition of monoclonal mouse anti-α-Gal antibody (mAb) M86 binding to α-Gal-BSA was measured by inhibition ELISA after incubating the mAb M86 with increasing concentrations of total proteins (5, 10 and 20 ng/ml) extracted from each *Plasmodium* species. Proteins extracted from wild-type and *ggta1* (α1,3GT) knockout (KO) *Sus scrofa* (pigs) kidney samples were used as positive and negative controls, respectively. α-Gal-BSA was used as an additional positive control. Results were expressed as percentage of inhibition (%). Experimental procedures are available in **Supplementary Material S1**.

**Figure 2:**
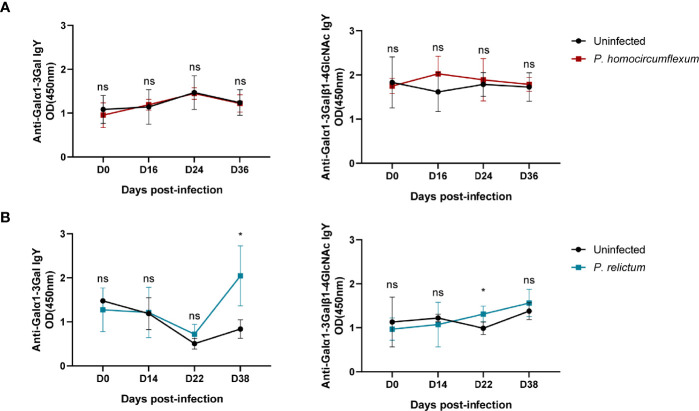
Anti-α-Gal Abs in sera samples of canaries experimentally infected with *Plasmodium* species. The levels of IgY against Galα1-3Gal and Galα1-3Galβ1-4GlcNAc in sera of canaries infected with *P. homocircumflexum* (COLL4) **(A)** or *P. relictum* (SGS1) **(B)** were measured by ELISA. The levels of anti-α-Gal IgY between groups were compared by one-way ANOVA with Dunnett’s multiple comparison test applied for individual comparisons (*p < 0.05, ns: not significant, 1 experiment for each *Plasmodium* species, n = 3 to 5 per group and three technical replicates per sample). Experimental procedures are available in **Supplementary Material S1**.

Our preliminary data shows the presence of α-Gal in protein extracts from three *Plasmodium* species, *P. ashfordi* (genetic lineage GRW2), *P. relictum* (SGS1) and *P. homocircumflexum* (COLL4), obtained from experimentally infected passerine birds, Eurasian siskins (*Carduelis spinus*) (**Figure 1**). Natural anti-α-Gal Abs have variable affinity for different α-Gal-related antigens, including Galα1-3Gal disaccharide and Galα1-3Galβ1-4GlcNAc trisaccharide. To test the immunogenicity of avian *Plasmodium* α-Gal, sera levels of IgY against Galα1-3Gal and Galα1-3Galβ1-4GlcNAc were measured by ELISA in canaries (*Serinus canaria domestica*) experimentally infected with *P. homocircumflexum* or *P. relictum*. The levels of circulating IgY against Galα1-3Gal and Galα1-3Galβ1-4GlcNAc did not change over time in birds infected with *P. homocircumflexum* (**Figure 2A**). In contrast, the levels of circulating IgY against Galα1-3Gal increased significantly at day 38 post infection with *P. relictum*, while the levels of Galα1-3Galβ1-4GlcNAc did not change (**Figure 2B**). These preliminary results showed the presence of α-Gal on avian malaria parasites, and demonstrated the immunogenicity of the *P. relictum* glycan in birds. It is thus plausible that anti-α-Gal IgY induced by blood stages of *P. relictum* mediate the opsonization (i.e., an immune process which uses opsonins such as Abs to tag foreign pathogens for elimination by phagocytes.) of blood parasitic stages. What caused the absence of anti-α-Gal Abs response in *P. homocircumflexum*-infected birds is unclear to us. It can be related with very low levels of the glycan in *P. homocircumflexum*. It is noteworthy that among the three *Plasmodium* species tested, *P. homocircumflexum* has the lowest levels of α-Gal (**Figure 1**).

## The Gut Microbiota and *Plasmodium* Infection

Studies on avian gut microbiota are scarce (33). According to Grond et al. (33), from 1980 to 2017 the number of published studies on mammals (including humans), poultry and wild birds microbiota were 16200, 1200 and 32, respectively. The underrepresentation of wild bird microbiota studies in the literature opens a gap in our understanding of the contribution of microbiota to host health, fitness, and immunity in metazoans. Most avian microbiota studies have focused on economically important species such as chicken and turkey (19). In wild birds, the research has focused on the identification of extrinsic and intrinsic factors affecting gut microbiota composition. Next-generation sequence analyses in different bird species have uncovered the diversity of microbial communities in gut microbiota (20). Core microbiota in avian enteric tract included the bacterial taxa Proteobacteria, Firmicutes, Fusobacteria, Actinobacteria and Bacteroidetes (20, 34). The first meta-analysis of the avian gut microbiota revealed that the dominant factor contributing to the composition of gut microbiota in wild birds is host taxonomic category (35). However, Grond et al. (34) showed that local environment has a large influence on gut microbiota composition of Arctic-breeding shorebirds. Another study on Brown-headed Cowbirds (*Molothrus ater*) showed that environmental factors, rather than genetics, influence passerine gut microbiota composition (36). The impact of other factors such as diet, probiotic treatment, kinship and captive rearing conditions have been assessed in poultry, but these may not be representative of wild life species (19). Ordination analyses of avian microbiota revealed that gastrointestinal tract microbial communities group according to sampling region (i.e., crop, caeca, cloaca, and fecal) (35), as in reptiles (37) and mammals (38). Parasite infection may also influence bird microbiota composition. For example, Coccidiosis, caused by *Eimeria* spp. infection in poultry affects the composition and integrity of gut microbiota, which in turn elevated susceptibility to other diseases (39).

A recent study by Videvall et al. (40) explored the impact of *Plasmodium* infection of the uropygial gland microbiota of house sparrows. Results showed differences in the abundance of certain bacterial genera according to *Plasmodium* infection status. These results suggest a possible interaction between infection and microbiota. Some microorganisms have been identified as potential symbionts in the uropygial gland (41). Based on functional predictions using 16S rRNA sequences, uropygial gland bacteria were suggested to produce metabolites with antimicrobial properties, such as terpenes (41). Whether microbes within the uropygial gland or intestinal tract play protective roles against avian malaria infection in birds is currently unknown.

However, strong evidence suggests a link between mammalian gut microbiota and human and murine malaria infection. Villarino et al. (42) found that differences in the gut microbiota determined severity of malaria caused by *P. yoelii* infection in mice. Further microbiota composition analysis revealed increased abundance of *Lactobacillus* and *Bifidobacterium* in resistant mice (42). In a follow up study, the authors tested whether synthesis of short-chain fatty acids (SCFAs), a well-studied mechanism by which the intestinal microbiota exerts an effect on host health explained the differences in susceptibility to malaria (43). However, the presence of fecal SCFAs did not explain differential susceptibility to *P. yoelli* infection (43). Microbial metabolites could influence the immune response to infection or alter parasite growth, as demonstrated by the impairment of *Salmonella* growth by the presence of propionate produce by Bacteroides (44). To gain further insight into the mechanism of resistance, another group determined the combined host and microflora metabolome and metatranscriptome (45). Differences in metabolite pools were associated with malaria resistance or susceptibility in mice (45). However, the relevance of identified metabolites for malaria infection, microbial community activity, or host response remains elusive (45). Another study demonstrated that both malaria infection severity and pregnancy outcome can by influenced by modulating the composition of the gut microbiota in an outbred mouse model for malaria in pregnancy (46).

Although the mechanism of microbiota-mediated resistance to murine malaria has not been elucidated, additional evidence further supports a protective role of host microbiota against malaria infection. For example, gut microbiota composition is very different between malaria endemic and non-endemic countries (47). Microbiota composition differences can be due to variation in diet, but also to host–pathogen adaptations, in which individual from endemic countries acquired, maintain and develop a gut microbiota that may influence protection to malaria transmission and/or tolerance to severe malaria (48). In agreement with this idea, observational studies from malaria-endemic regions show correlations between the composition of gut microbiota and lower risk of malaria infection (26).

It is important to mention that in the studies mentioned above by Villarino et al. (42), Chakravarty et al. (43) and Stough et al. (45), a mechanism mediated by anti-α-Gal Abs was rule out, as the experiments were carried out in wild-type mice that express α-Gal and cannot produce anti-α-Gal Abs. This suggests that different mechanisms may account for malaria resistance in animals that produce endogenous α-Gal (e.g., wild-type mice) and those that do not produce α-Gal (e.g., humans and birds). An interesting observation is that bacteria of the family Enterobacteriaceae (e.g., *Escherichia*-*Shigella*) and the genus *Bifidobacterium* were found in the microbiota of Malian children with lower risk of *P. falciparum* infection (49). The protective mechanism mediated by *Bifidobacterium* might be activated in animals that produce (e.g., wild-type mice), or not (e.g., humans), endogenous α-Gal. However, the presence of Enterobacteriaceae expressing α-Gal may be an additional protective barrier against malaria infection in humans and birds.

## Enterobacteriaceae, a Rich Source of α1,3GT Genes in Human and Bird Microbiota

A recent study found 193 species and strains of bacteria containing α1,3GT genes in the human gut microbiota (9). Bacteria of the families Enterobacteriaceae (genus *Escherichia*-*Shigella*), Pasteurellaceae (genus *Haemophilus*), Lactobacillaceae (genera *Pediococcus, Lactobacillus*) are among those containing α1,3GT genes in the human gut microbiota. Among the α1,3GT genes identified in the human microbiome are the *gspA*-general secretion pathway protein A (accession K02450), *waaL*, *rfaL*-O-antigen ligase (K02847); *waaO*, *rfaI*-UDP-glucose: (glucosyl) LPS alpha-1,3-glucosyltransferase (K03275); *waaJ*, *rfaJ*, UDP-glucose: (galactosyl) LPS alpha-1,2-glucosyltransferase (K03279); *waaR*, *waaT*, *rfaJ*- UDP-glucose/galactose: (glucosyl) LPS alpha-1,2-glucosyl/galactosyltransferase (K03276) and *waaI*, *rfaI*-UDP-D-galactose: (glucosyl) LPS alpha-1,3-D-galactosyltransferase (K03278).

**Figure 3:**
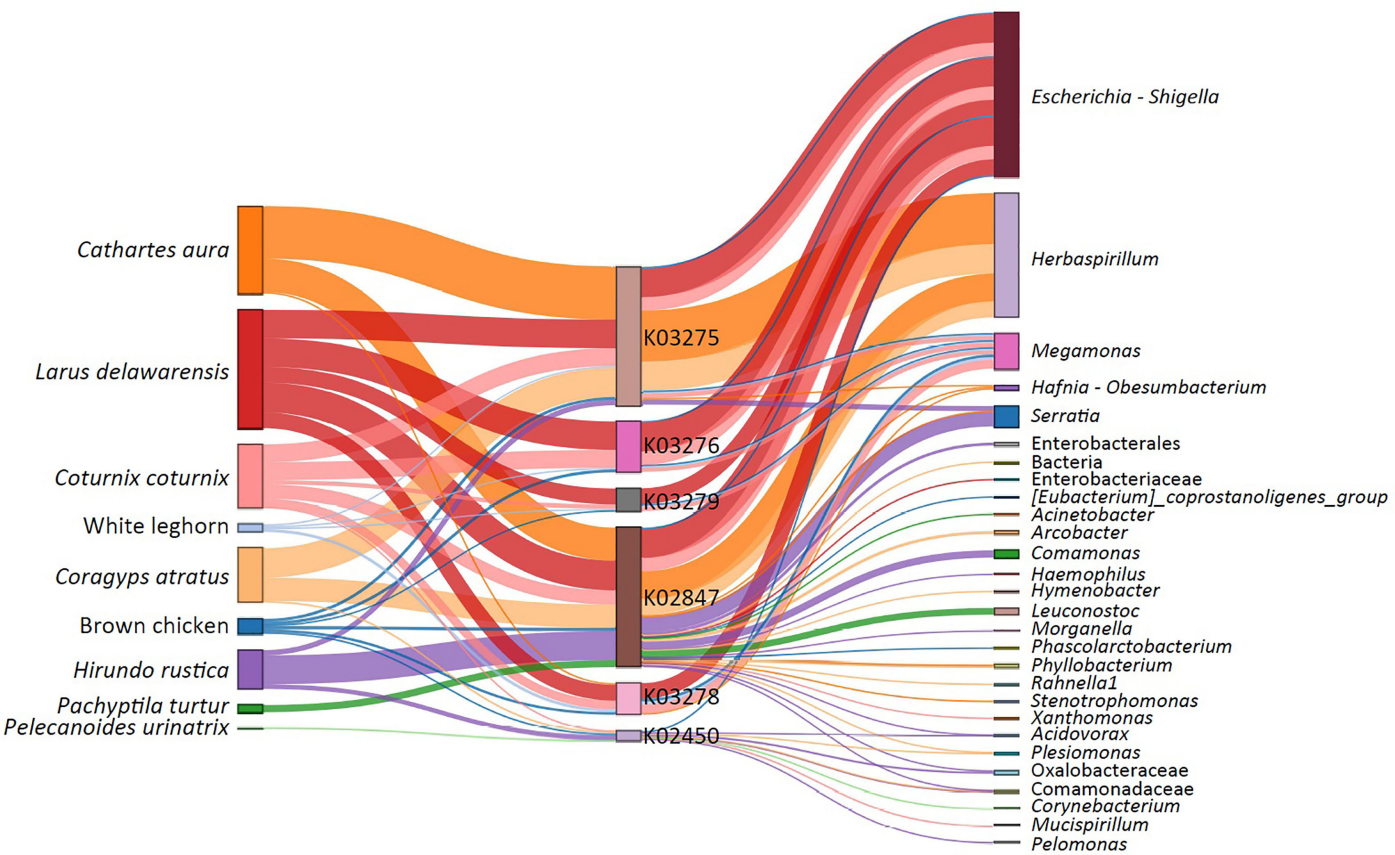
Contribution of commensal bacteria to α1,3GT genes in the microbiome of wild birds and poultry. Alluvial plot showing the presence α1,3GT genes (i.e. K02450, K02847, K03275, K03279, K03276, K03278) in the microbiome from various avian host. The bacterial genera harboring these genes in each host are also displayed. The presence and abundance of α1,3GT genes was inferred from the 16S rRNA data using the bioinformatics pipeline PICRUSt2 for metagenome prediction. The *α1,3GT* genes were annotated based on KEGG orthologs (KO) database as reference. K02450: *gspA*, general secretion pathway protein A; K02847: waaL, rfaL, O-antigen ligase [EC:2.4.1.-]; K03275: *waaO*, *rfaI*, UDP-glucose:(glucosyl)LPS alpha-1,3-glucosyltransferase [EC:2.4.1.-]; K03279: waaJ, rfaJ, UDP-glucose:(galactosyl)LPS alpha-1,2-glucosyltransferase [EC:2.4.1.58]; K03276: *waaR*, *waaT*, *rfaJ*, UDP-glucose/galactose:(glucosyl)LPS alpha-1,2-glucosyl/galactosyltransferase [EC:2.4.1.-]; K03278: *waaI*, *rfaI*, UDP-D-galactose:(glucosyl)LPS alpha-1,3-D-galactosyltransferase [EC:2.4.1.44]. Node segments by columns represent host (First column), functional genes (Second column) and bacterial taxa (Third column), respectively. Node size is proportional to the abundance of contributing host or bacterial taxa or genes. The cords indicate the connections between host, the α1,3GT genes and taxa. The contribution of each taxon to different α1,3GT genes is represented proportionally by the size of cords. Experimental procedures are available in **Supplementary Material S1**.

**Figure 4:**
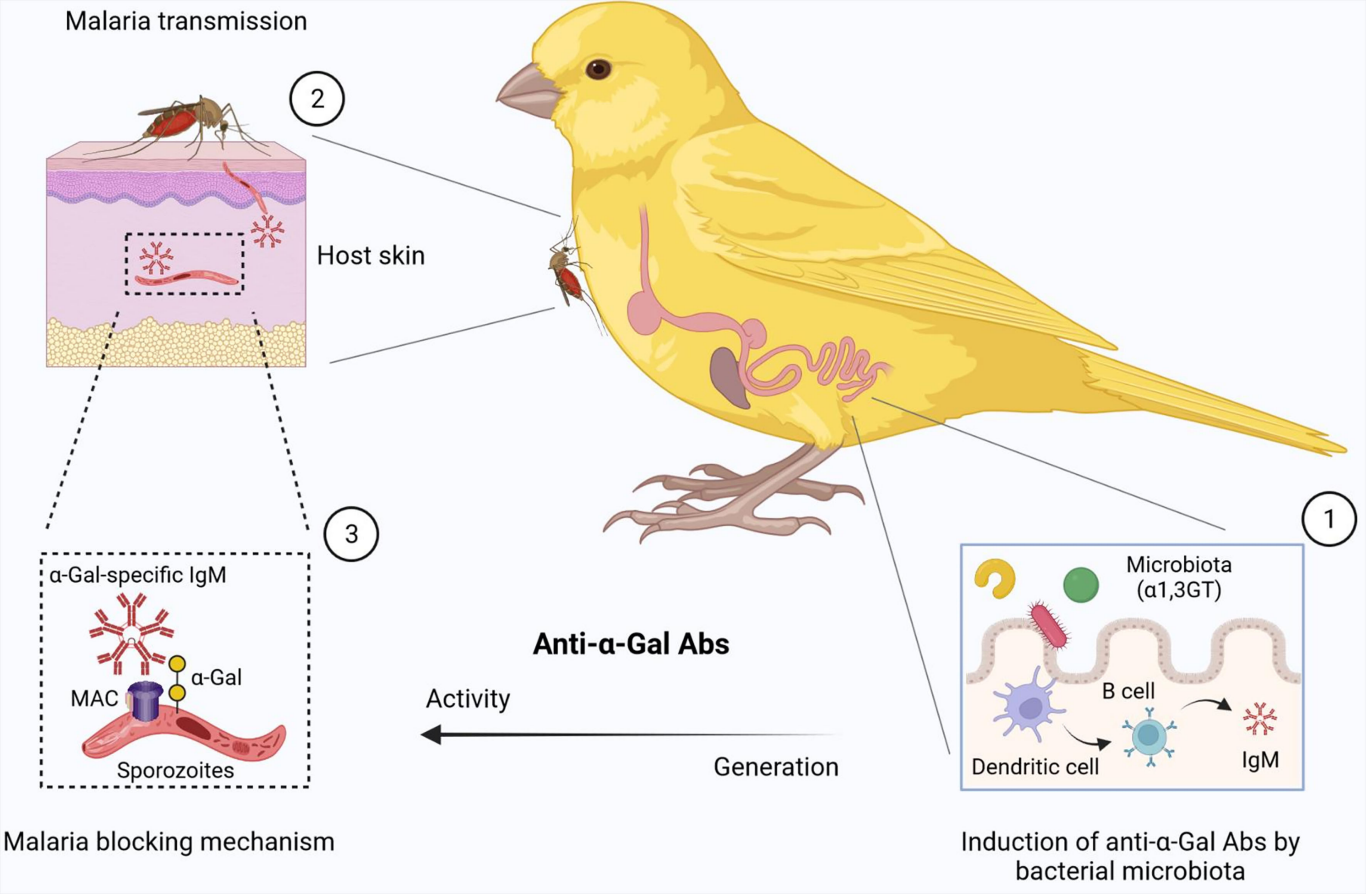
Proposed malaria blocking mechanism mediated by gut microbiota in birds (1). The α-Gal-expressing microbes cause the stimulation of B cells to produce anti-α-gal IgM. The structure of the α-Gal glycan is similar in microbiota and *Plasmodium* sporozoite surface. (2) *Plasmodium* sporozoite transmission by mosquitoes, (3) can be blocked *via* a complement-mediated lysis of sporozoites in the skin. MAC, Membrane Attack Complex. Figure created with BioRender.com.

We hypothesize that different migratory behaviors (i.e., non-migrating and short or long-distance migrants), diets (i.e., insectivorous and seedeaters) and ecology conditions might influence gut microbiota composition, as well as the distribution of α1,3GT genes in bacterial microbiota of birds. Here we predicted the presence of α1,3GT genes (i.e., K02450, K02847, K03275, K03279, K03276, K03278) in the microbiomes from various wild bird species representing diverse diets and habitats. These included the Japanese quail (*Coturnix coturnix*), fairy prion (*Pachyptila turtur*), common diving petrel (*Pelecanoides urinatrix*), barn swallow (*Hirundo rustica*), gulls (*Larus delawarensis*), black vulture (*Coragyps atratus*), turkey vulture (*Cathartes aura*), and two breed of poultry (*Gallus gallus domesticus*), white leghorn and brown chicken. The results showed that α1,3GT genes were distributed in more than 140 bacterial taxa in the microbiome of the analyzed birds. Not all taxa contributed equally to the distribution of α1,3GT genes, as various of these taxa had very low abundance in the microbiomes and thus contributed only marginally to α1,3GT genes. The set of taxa with the highest abundance and contribution (~ 20% of the total number of taxa) was selected for further analysis. All the α1,3GT genes were present in all the bird species under study, except for *P. turtur* and *P. urinatrix* in which only the genes K03278 and K02450 were found, respectively (**Figure 3**). Among these genes, the most frequent were K03275 and K02847. Notably, the taxa with the highest contribution to α1,3GT genes in these birds was Enterobacteriaceae (genus *Escherichia*-*Shigella*, **Figure 3**), as in the human microbiome (9). Other bacterial genera such as *Herbaspirillum, Megamonas* and *Serratia* also contain α1,3GT genes (**Figure 3**). This preliminary data suggests that birds are an ideal model to test the relation between gut microbiota, *Plasmodium* infection and anti-α-Gal immunity. Based on the above evidence, a malaria blocking mechanism mediated by anti-α-Gal IgM in birds is proposed (**Figure 4**).

## Host Gut Microbiota Manipulation and Anti-α-Gal Immune Response

A recent study demonstrated for the first time that modulation of anti-α-Gal immunity using gut microbiota manipulation protects birds against avian aspergillosis, caused by experimental infection with *Aspergillus fumigatus*, a pathogen expressing α-Gal (7). Specifically, oral administration of *E. coli* O86:B7 increased the levels of IgY against the disaccharide Galα1-3Gal, along with a decrease in the levels of IgY against the trisaccharide Galα1-3Galβ1-4GlcNAc in sera of treated turkeys. Oral administration of *E. coli* O86:B7 was also associated with decreased anti-α-Gal IgA in lungs compared with non-treated turkeys. Interestingly, decreased levels of anti-α-Gal IgA were accompanied by a reduction in the occurrence of lung granulomas, which is associated with acute aspergillosis in turkeys. These results suggest a crosstalk mechanism in birds by which the gut microbiota modulates the immune response in the lungs (50). In this infection model (i.e., intratracheal infectious challenge with *A. fumigatus*) (7), the mechanism of protection against avian aspergillosis does not seem mediated by increased anti-Galα1-3Gal IgY in sera. However, increased sera levels of anti-Galα1-3Gal IgY induced by oral administration of *E. coli* O86:B7 may be relevant to prevent avian malaria transmission by mosquitoes.

Other bacterial species such as *Aeromonas veronii* and *Pseudomonas entomophila* have been used to modulate the microbiota and induced anti-α-Gal immunity (18). Recently, Pacheco et al. (18) reported that native *A. veronii* and *P. entomophila* bacteria isolated from the zebrafish gut have high content of α-Gal. Fish fed with commercial feed coated with *A. veronii* or *P. entomophila* showed increased anti-α-Gal IgM levels. Anti-α-Gal IgM response in the *P. entomophila* group was associated with a significant reduction in mycobacterial infection, caused by *Mycobacterium marinum* (18). These results provide evidence that various bacteria species with high α-Gal content can be identified in the gut microbiota of animals lacking endogenous α-Gal. Probiotic treatment in zebrafish was also associated with significant changes in the beta diversity and taxa abundance in fish microbiota (18). Furthermore, the abundance of some taxa was negatively correlated with the anti-α-Gal IgM levels, suggesting a role of anti-α-Gal Abs in modulating fish gut microbiota, as reported for mammalian gut microbiota (51). Another interesting insight of the zebrafish study is that gene expression analysis in probiotic-treated fish challenged with *M. marinum* suggests that protective mechanism associated with anti-α-Gal immunity can go beyond anti-α-Gal Abs-mediated control of mycobacteria (18). Protection was also associated with B-cell maturation, induced innate immune responses and beneficial effects on nutrient metabolism and oxidative stress (18). The results of this trial in fish support immune and metabolic implications of α-Gal-mediated immunity, beyond induction of anti-α-Gal Abs. It remains to be tested whether α-Gal-mediated immunity induced by oral administration of *E. coli* O86:B7, or other bacteria expressing α-Gal, produce malaria resistance associated with broad metabolic and immune effects in birds.

## Crosstalk Between Host and Vector Gut Microbiota

The presence and distribution of α1,3GT genes in bacterial microbiota suggests the production of natural anti-α-Gal Abs in wild birds. Can the anti-α-Gal Abs bind and/or lyse bacteria expressing α-Gal in the mosquito microbiota? What impact would mosquito microbiota modulation by anti-α-Gal Abs have on mosquito fitness and/or malaria transmission? Host antibodies taken in the blood meal can target pathogens and bacterial microbiota within hematophagous arthropods (52). Functional host antibodies have been shown to interact with symbionts in *Rhodnius prolixus* (53) and *Glossina morsitans* (54) as well as with bacterial microbiota in mosquitoes (55) and ticks (56, 57). Recent research showed that α1,3GT genes are broadly distributed in tick bacterial microbiota (56). Immunization with a tick microbiota Enterobacteriaceae, caused significant mortality of engorging ticks (56). Anti-α-Gal IgM and IgG were associated with a mean mortality of approximately 45% in ticks fed on α1,3GT-deficient mice (56). Anti-microbiota vaccine directed at Enterobacteriaceae in the microbiota of *I. scapularis* disrupted both the makeup and functions of the microbiome and decreased pathways central to lysine degradation (57). Interestingly, Enterobacteriaceae (i.e., *Escherichia*-*Shigella*) is shared by the microbiota of ticks and mosquitoes, but not of sandflies (58). Anti-microbiota vaccines are a microbiome manipulation tool for the induction of infection-refractory states in the vector microbiome (52). The evidence suggests that ingestion of avian anti-α-Gal Abs with the blood meal could target mosquito microbiota expressing α-Gal (e.g., Enterobacteriaceae) with a potential impact on *Plasmodium* colonization of mosquito tissues.

## From Mother to Offspring: Transgenerational *vs.* Inter-Generational Immune Mechanisms

Evolution of complex traits such as host resistance to pathogen infection can be linked to phenotypic variation due to ‘transgenerational’ or ‘inter-generational’ immune mechanisms (59, 60). ‘Transgenerational’ here is defined as transmission across generations (i.e., from grandparents to a grandchild), without involving direct exposure to the environmental stimulus that triggered the primal host response (60, 61). Unequivocal transgenerational transmission of an adult phenotype through the germ-line requires assessment of the F3 generation for embryonic exposure, and F2 generation for postnatal exposure (60). ‘Inter-generational’ represents the transmission of traits from one generation to the next (i.e., from mother to offspring). Phenotypic traits acquired by parents can be transmitted by transgenerational or inter-generational epigenetic inheritance (59, 61).

Epigenetic inheritance involves stimuli-triggered changes in gene expression due to processes that arise independent of changes in the underlying DNA sequence. Some of these processes include DNA methylation (62), histone modifications and/or chromatin-remodeling proteins (63). Epigenetic inheritance remains poorly explored in birds (64). However, some transgenerational epigenetic mechanisms have been described in birds (64, 65). For example, the regulation of immunoglobulin gene expression in the offspring of broiler hens under different environmental stimulus (i.e., unpredictable or predictable light regimens) was linked to epigenetic mechanisms (65, 66). These genes are involved in both neural development (67) and immunity, and their regulation suggests that immune parameters can be epigenetically transferred to the next generation (66). Although the epigenetic markers associated with this trait in birds remain to be identified. In addition to epigenetic inheritance, maternal antibodies have also been described as non-genetic, information-bearing molecules that transfer information about the immunologically relevant environment (e.g., exposure to pathogens) gathered by the mother to her offspring (65). The hypothesis of transfer of anti-α-Gal IgY with anti-malaria effect in birds can be tested without accounting for transgenerational epigenetic mechanisms.

## Inter-Generational Transmission and Avian Microbiota Reprogramming by Anti-α-Gal Abs

Several studies show that the inter-generational transfer of maternal Abs provides humoral immune defense against pathogens in eggs and early-life offspring. This mechanism is crucial as endogenous production of Abs in chicks occur only 10-14 days post-hatching (68). For example, Grindstaff et al. (69) reported that the offspring from female pied flycatchers (*Ficedula hypoleuca*) injected with *Salmonella typhimurium* lipopolysaccharide (LPS) had higher Abs levels compared to offspring from pied flycatchers not injected to this bacterial antigen. Kittiwakes (*Rissa tridactyla*) mothers naturally exposed to the tick-borne bacteria *Borrelia burgdorferi* transfer anti-*Borrelia* Abs to their eggs and offspring (70, 71). In addition, the levels of anti-*Borrelia* Abs of kittiwake chicks at 10 and 20 days of age were higher when 5 days old had significantly higher anti-*Borrelia* Ab titres (72). High anti-*Borrelia* Ab levels in 5-day-old chicks were presumably transferred from the mother (72). These results suggest that maternal exposure to pathogens, including vector-borne pathogens such as *Borrelia* or *Plasmodium*, can enhance the humoral immunity of early-life offspring.

**Figure 5:**
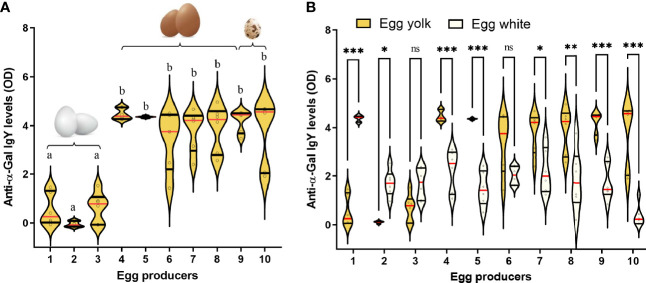
Anti-α-Gal Abs in bird eggs. **(A)** Anti-α-Gal IgY levels were measured by indirect ELISA in egg yolks of ten individual eggs. **(B)** The anti-α-Gal IgY levels in egg yolks are shown together and those in egg whites from the same eggs. The eggs were purchased from three (1-3, Leghorn hens), six (4-9, ISA brown hens) and one (10, quails) different commercial vendors in France. The levels of anti-α-Gal IgY between groups were compared by one-way ANOVA with Dunnett’s multiple comparison test applied for individual comparisons. Different letters in panel **(A)** indicate statistically significant differences (*p* < 0.05). In panel **(B)**, significant differences were denoted as *p < 0.05, **p < 0.001, ***p < 0.0001, ns: not significant. In both panels (n = 3 eggs per vendors and two technical replicates per sample). Experimental procedures are available in **Supplementary Material S1**.

The levels of anti-α-Gal IgY in eggs are variable (73, 74, **Figure 5A**), and they are more abundant in egg yolks than in egg whites (73, 74, **Figure 5B**). Anti-α-Gal IgY isolated from birds are able to bind α-Gal antigens in mammal tissues. Particularly, binding of avian anti-α-Gal Abs block the binding of human anti-α-Gal to xenograft endothelial cells (75, 76). Avian anti-α-Gal also block human blood complement activation and antibody-dependent cell-mediated lysis mechanisms that are responsible of hyperacute rejections in xenografts (75, 76). This shows the functionality of avian anti-α-Gal Abs. Whether anti-α-Gal Abs transmitted from the mother to egg to chick, have protective functions against malaria or other infectious diseases remains an open question.

Besides the preferential presence of IgY in egg yolks, IgM and IgA are predominantly found in egg whites (77). Difference on the distribution of Abs in different egg compartments could be associated to their immunity role in different part of bird’s body. While IgY is transported to the embryonic circulation, IgM and IgA are transferred to the gastrointestinal gut of developing chick, where they exert an important role in local immunity (77–80). IgA recognize bacteria in the gut microbiota (81–83) shaping the microbiota composition, and ecology, by limiting bacterial growth (84), or promoting bacterial interactions with the host, favoring bacterial retention, fitness, and colonization (85, 86). Removal of α1,3GT activity shaped the composition of the gut microbiota in mice (51). This occurred *via* an IgA-dependent mechanism, associated with targeting of α-Gal-expressing bacteria by IgA in mice (51). *Ggta1* deletion enhances microbiota-specific IgA responses without interfering with total IgA (51). Interestingly, the microbiota composition diverged between *ggta1* ^+/+^ and *ggta1* ^-/-^ mice in F3, F4 and F5 generations, suggesting that the α1,3GT-negative genotype per se alters microbiota composition (51), which suggest a transgenerational effect. Differences in avian anti-α-Gal IgA levels in eggs may be associated with variations in the gut microbiota of chicks, with reduced or increased abundance of defined subsets of species within bacterial families expressing α-Gal such as Enterobacteriaceae. IgA-mediated selection of commensal Enterobacteriaceae within the avian enteric microbiota would provide α1,3GT activity, high levels of circulating anti-α-Gal IgY and IgM with potential malarial resistance effects.

## Exploring the Ecology of the Bird-Malaria-Microbiota System

To test our hypothesis, we propose firstly to perform an observational field study and sample wild bird species with different migratory behaviors, habitats and diets. Then, to measure the levels of natural anti-α-Gal Abs in different bird species and the presence of α-Gal in different *Plasmodium* spp. which can help elucidating whether an association exists between microbiome composition, the levels of anti-α-Gal Abs and *Plasmodium* infection within and between habitats and diets. The immunological analysis of samples obtained from wild birds will provide valuable information about the changes in anti-α-Gal Abs in infected birds within the same habitat and the impact of haemosporidian parasites on the immune system of different host species.

Our preliminary results from three different *Plasmodium* species gives a hint that different malarial parasites exhibit different levels of α-Gal in protein extracts. We expect that the α-Gal levels vary in *Plasmodium* species, especially from different, distantly related, subgenera, as these parasites would have some developmental differences during the life cycle and different life history traits (87). For instance, *P. ashfordi* (GRW2), a tropical generalist parasite infecting 21 different bird species (24) from *Novyella* subgenus, showed the highest level of α-Gal (**Figure 1**), while other two parasites exhibited relatively lower levels. Further analysis of different parasites with various specializations can help untangling the questions related to infectivity of avian malarial parasites.

## From Observation to Ecology-Informed Experimentation

**Figure 6:**
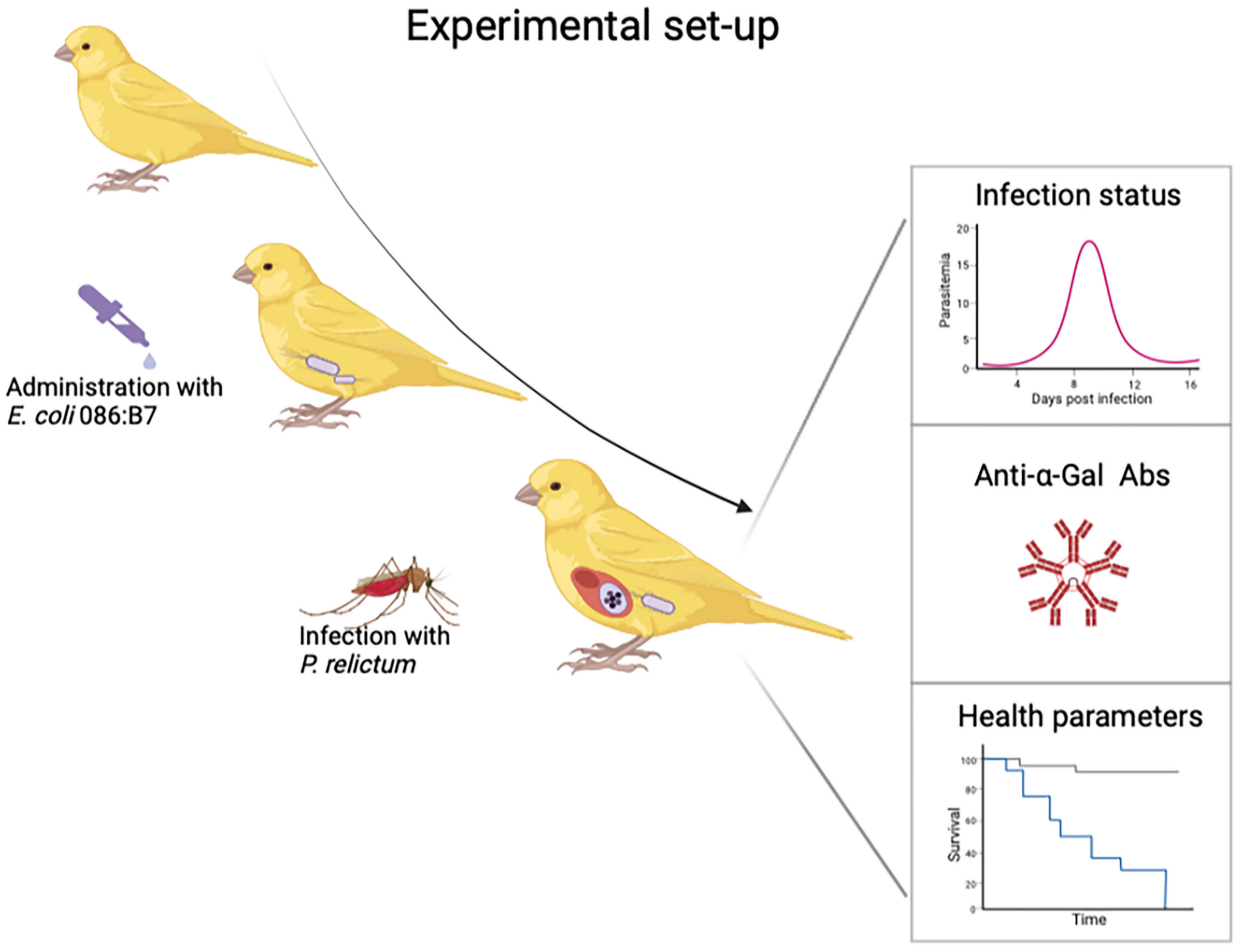
Experimental set-up to test whether orally administered bacteria expressing high levels of α-Gal lead to increase of anti-α-Gal Abs and blocks transmission of *P. relictum*. Figure created with BioRender.com.

To test the impact of anti-α-Gal Abs on the infectivity of malarial parasites, experimental studies with canaries, malarial parasites, gut bacteria and mosquitoes can be performed. We will use avian malarial parasite *P. relictum*, which is listed among the most invasive organisms in the world, infecting more than 300 bird species and is prevalent all around the world (88). *Culex pipiens* mosquitoes, the natural vector of *P. relictum*, will be used as a vector. *Escherichia coli* O86:B7 will be used as a bacterial source of α-Gal. This system will give us a unique possibility to experimentally test whether the gut microbiota bacteria expressing α-Gal increases anti-α-Gal Abs with an impact on the infectivity of malarial parasites (**Figure 6**). We predict that avian malaria can be affected by increased anti-α-Gal Abs when small numbers of sporozoites are inoculated. Partial infections of hosts with avian malarial parasites were reported in previous experimental studies (89, 90), where only some *Plasmodium* parasites developed parasitemia after mosquito bite. Factors which could influence the survival of sporozoites and therefore infectivity of the host were studied in many previous studies (91–94), however, none of them evaluated possible impact of the microbiota.

Experimental study with mothers having different α-Gal immunity status and comparison of offspring resistance to malarial pathogen, will allow assessment of the importance of the microbiome and anti-α-Gal Abs on infectivity of early life offspring with vector-borne pathogens. According to ecological studies, nestlings are less infected with haemosporidian parasites (25, 95, 96). However, in some species of diurnal raptors and owls, nestlings can be infected with *Leucocytozoon* spp. (species closely related to *Plasmodium*) up to 30% or even 70% (25). Apparently, some differences of nestling infectivity could be explained by the prolonged time stay of hatched nestlings in the nest (25). Experimental study with chaffinches showed that nestlings up to 12 days of age usually do not get infected with haemosporidian parasites, while birds of the same species of approximately one-month age exhibit up to 36% prevalence of haemosporidian infection (25). These differences probably could also be explained by inter-generational defense against pathogens, which is formed in offspring after immunization of mother before lying eggs (69). Our experimental system will enable to answer if the protective effect mediated by anti-α-Gal Abs against malaria can be passed from parents to offspring as a mechanism of inter-generational immunity and will help understanding the level of protective shield against infection diseases in birds.

## Discussion

The maintenance and functionality across time and space of the complex populations of microbes present in the animal intestine is poorly understood. Birds have a global distribution, being in every continent and exhibiting an extreme morphological (97) and ecological diversity (98). They have different diets, from strictly carrion to nectar feeders, with corresponding variation in intestinal morphology. Among other factors (20), bird microbiota composition can be influenced by host genetics (99), host phylogeny, location within the gut (35), diet (19, 35), and association with humans (35). Little is known, however, about how microbiota diversity under these factors influence susceptibility or resistance to avian diseases, or whether certain microbiota assemblies are under selective pressures by parasites in natural systems. The unparalleled genetic and rich phenotypic diversity of avian malaria pathogens, together with variations in infectivity of avian malarial parasites provides endless opportunities for exploring how bird microbiota contributes to the selective pressures under which hosts and parasites evolve. This makes bird-malaria-microbiota interactions a unique system to understand the impact of microbiota on animal ecology.

**Figure 7:**
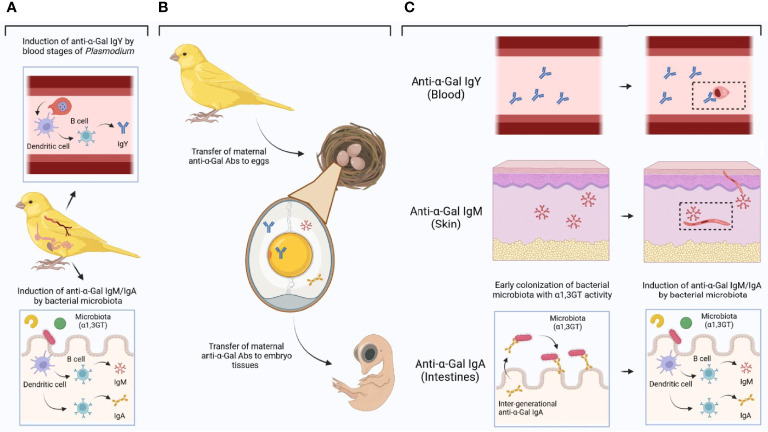
Induction of anti-α-Gal Abs in birds and tissue-specific mechanisms of protection. **(A)** Blood stages of some malaria parasites induces anti-α-Gal IgY, while bacterial microbiota with α1,3GT activity could induce anti-α-Gal IgM/IgA. **(B)** Anti-α-Gal IgY, IgM and/or IgA can be transferred from mother to eggs and from there to the embryo and subsequently to the chick. **(C)** Different isotypes of anti-α-Gal Abs could be located in different embryo/chick tissues (tissue blocks to the left) with tissue-specific functions (tissue blocks to the right). Anti-α-Gal IgY could mediate opsonization of blood stages of malaria parasites (the insert represents an anti-α-Gal IgY-*Plasmodium* complex). Anti-α-Gal IgM could target sporozoites in the skin (the insert represents complement-mediated lysis of *Plasmodium* upon anti-α-Gal IgM binding on the surface of the parasite). Anti-α-Gal IgA could be involved in the early colonization of bacterial microbiota with α1,3GT activity with the subsequent activation of anti-α-Gal IgM/IgA production mechanisms. Figure created with BioRender.com.

Evidence shows that bacterial communities in birds are inherited (19, 99), which suggest that microbiota composition can be under selection. The spread of highly virulent avian malaria infections across a bird population (100) could select for individuals carrying a protective microbiome. This is, if some microbiome composition decreases the susceptibility to malaria infection in birds, as in human and rodent malaria (see above ‘The gut microbiota and *Plasmodium* infection’). The research results revised and summarized here, together with our preliminary data, supports the hypothesis that avian resistance to malaria is influenced by variations in α1,3GT activity in bird gut microbiota which in turn elicits anti-α-Gal Abs with anti-malaria activity. The evidence presented and discussed here further supports that gut microbiota triggers anti-α-Gal IgM and/or IgA, while blood stages of some *Plasmodium* species can triggered anti-α-Gal Abs IgY in birds (**Figure 7A**). These three isotypes of anti-α-Gal Abs can be transfer from mother to offspring (**Figure 7B**), providing malaria resistance mechanisms in a tissue-specific manner (**Figure 7C**).

The field and experimental studies proposed here can be used to test specific questions to demonstrate this hypothesis such as: (*i*) does the enteric microbiome of birds influences the resistance to malaria *via* anti-α-Gal IgM and/or IgY? (*ii*) are anti-α-Gal IgM and IgY in the eggs associated with early resistance to malaria in chicks? and (*iii*) do anti-α-Gal IgA in eggs can favors the colonization of bacteria with α1,3GT activity such as Enterobacteriaceae in the gut microbiome of newly hatched chicks? Avian malaria is the oldest experimental system for investigating the biology and transmission of *Plasmodium* parasites (101). In the light of the new hypothesis and theories presented here, this ‘old model’ can shed light on the ecological impact of microbiota diversity and the role of anti-α-Gal Abs in malaria resistance.

## Data Availability Statement

Publicly available datasets were analyzed in this study. This data can be found here: Published and publicly available 16S datasets were used for the prediction of functional traits of the microbiome of wild birds and poultry. Datasets of eleven untreated wild birds were included in this study: Japanese quail from Kohl et al., 2008 (accession PRJNA244306), fairy prion and common diving petrel from Dewar et al., 2014 (PRJEB1549), barn swallow from Kreisinger et al., 2015 (PRJEB7057), gulls from Koskey et al., 2013 (PRJNA229760), black and turkey vultures from Roggenbuck et al., 2014 (PRJNA243051). Finally, two datasets from two breeds of poultry were also included: untreated brown chicken (Hy-line brown) from Xu et al., 2019 (PRJNA510025) and white leghorn (Hy-Line W36) and brown chicken (Hy-line brown) reared in conventional cages from Adhikari et al., 2020 (PRJNA627663).

## Ethics Statement

All procedures were performed at the Nature Research Centre in Vilnius, Lithuania, according to Lithuanian and International Guiding Principles for Biomedical Research Involving Animals (2012). Infection experiments and other procedures were reviewed and approved by the Lithuanian State Food and Veterinary Service, Ref. No. 2020/07/24-G2-84 and International Research Cooperation Agreement between the Biological Station “Rybachy” and the Nature Research Centre (25/05/2010, 04/09/2015). The assessment of the animal health and all described procedures were implemented by trained professionals (under licenses 2012/02/06-No-208, and 2016/01.29-No-344).

## Author Contributions

AC-C and VP conceived the study. LM-H, AW-C, VP, and JA performed the experiments and acquired the data. DO, AW-C, LM-H, and AC-C analyzed the data. DO, AW-C, and AC-C prepared figures. AC-C, VP, and JF contributed reagents and other resources. AC-C, VP, and DO supervised the work. AC-C, LM-H, and VP drafted the first version of the manuscript. All the authors made editorial contributions and revised and accepted the final version of the manuscript.

## Funding

UMR BIPAR is supported by the French Government’s Investissement d’Avenir program, Laboratoire d’Excellence “Integrative Biology of Emerging Infectious Diseases” (grant no. ANR-10-LABX-62-IBEID). AW-C is supported by Programa Nacional de Becas de Postgrado en el Exterior “Don Carlos Antonio López” (grant no. 205/2018).

## Conflict of Interest

The authors declare that the research was conducted in the absence of any commercial or financial relationships that could be construed as a potential conflict of interest.

## Publisher’s Note

All claims expressed in this article are solely those of the authors and do not necessarily represent those of their affiliated organizations, or those of the publisher, the editors and the reviewers. Any product that may be evaluated in this article, or claim that may be made by its manufacturer, is not guaranteed or endorsed by the publisher.
