# Supplementary material for: Exploring the Ecological Implications of Microbiota Diversity in Birds: Natural Barriers Against Avian Malaria
Source: Front Immunol. 2022 Feb 17;13:807682. doi: 10.3389/fimmu.2022.807682 (PMC8891477; doi:10.3389/fimmu.2022.807682)
Supplement: Supplementary file 1 [file DataSheet_1.pdf]

Supplementary material S1

Exploring the ecological implications of microbiota diversity in birds: natural barriers against avian malaria

Vaidas Palinauskas[1,†,*] , Lourdes Mateos-Hernandez[2,†], Alejandra Wu-Chuang[2], Dasiel Obregon[3], Jose de la Fuente[4,5], Jusė Aželytė[1], Alejandro Cabezas-Cruz[2,*]

[1] Nature Research Centre, Akademijos 2, Vilnius, LT-09412, Lithuania
[2] ANSES, INRAE, Ecole Nationale Vétérinaire d'Alfort, UMR BIPAR, Laboratoire de Santé Animale, Maisons-Alfort, F-94700, France.
[3] School of Environmental Sciences University of Guelph, Guelph, Ontario N1G 2W1, Canada.
[4] SaBio, Instituto de Investigación en Recursos Cinegéticos IREC-CSIC-UCLM-JCCM, Ronda de Toledo 12, 13005 Ciudad Real, Spain
[5] Department of Veterinary Pathobiology, Center for Veterinary Health Sciences, Oklahoma State University, Stillwater, OK 74078, USA

† Equal contribution

*** Correspondence:** Alejandro Cabezas-Cruz (alejandro.cabezas@vet-alfort.fr) and Vaidas Palinauskas (palinauskas@gmail.com)

Materials and methods

Identification of α-1,3-galactosyltransferase genes in bird microbiome

Original 16S datasets

Published and publicly available 16S datasets were used for the prediction of functional traits of the microbiome of wild birds and poultry. Datasets of eleven untreated wild birds were

included in this study: Japanese quail from Kohl et al. (1) (BioProject accession: PRJNA244306), fairy prion and common diving petrel from Dewar et al. (2) (PRJEB1549), barn swallow from Kreisinger et al. (3) (PRJEB7057), gulls from Koskey et al. (4) (PRJNA229760), black and turkey vultures from Roggenbuck et al. (5) (PRJNA243051). Finally, two datasets from two breeds of poultry were also included. Brown chicken (Hy-line brown) from Xu et al. (6) (PRJNA510025) and white leghorn (Hy-Line W36) and brown chicken (Hy-line brown) reared in conventional cages from Adhikari et al. (7) (PRJNA627663).

Processing of original raw sequences and prediction of functional traits

Raw sequences of wild birds and poultry were downloaded from SRA repository (8). Paire-end or single-end raw sequences were downloaded and de-interlaced using the SRA-Toolkit (http://ncbi.github.io/sra-tools/). However, to standardize the analytical procedures, only the forward (single-end) sequences of each dataset were used. All datasets were then processed similarly, using QIIME2 software (v. 2021.4) (9). The fastq files were trimmed, filtered based on the quality score associated to each nucleotide in each read and merged using DADA2 software (10) implemented in QIIME2. After, reads were denoised into amplicon sequence variants (ASVs) and taxonomy was assigned to ASVs using a classify sklearn naïve Bayes taxonomic classifier (11) based on SILVA database (release 138) (12). The 16S ASVs package from each dataset were then used for metagenome predictions, using the PICRUSt2 pipeline (13). Briefly, ASVs were placed into a reference tree containing thousands of prokaryotic genomes, which is used to infer gene family copy numbers from ASVs dataset. The predictions were based on Kyoto Encyclopedia of Genes and Genomes (KEGG) orthologs (KO) (14). Our analyses were focused on the detection of α-1,3-galactosyltransferase genes, specifically on the identification of the bacterial taxa harboring these genes.

Determination of anti-α-Gal antibodies in eggs

Indirect ELISA for relative quantification of anti-α-Gal IgY levels in eggs

Eggs were purchased from three (Leghorn hens), six (ISA brown hens) and one (quails) different commercial vendors in France. To measure the levels of anti-α-Gal IgY in eggs, an

indirect ELISA was performed as previously described (15), with some modifications. 96-wells ELISA plates (Thermo Scientific, Waltham, MA, USA) were coated with α-Gal linked to Human Serum Albumin (α-Gal-HSA; Dextra Laboratories, Reading, UK) 50ng/wells in buffer carbonate/bicarbonate (0.5M, Ph: 9.6) and incubated overnight at 4 °C. Wells were washed three times and blocked with 100 µL of 1% HSA/PBS for 2 h at room temperature (RT). After three washes, egg yolk and egg white samples diluted in PBS (1:50), were added and incubated for 1 h at 37 °C in shaking. Plates were washed three times and HRP-conjugated antibodies (Abs) goat anti-chicken IgY (Sigma-Aldrich, St. Louis, MO, USA) were added at 1:1500 dilution PBS (100 µL/well) and incubated for 1 h at RT. The plates were washed three times and the reaction was developed by adding 100 µL ready-to-use TMB solution (Promega, Madison, WI, USA) at RT for 20 min in the dark, and then stopped with 50 µL of 0.5 M H_2SO_4. The OD was measured at 450 nm using an ELISA plate reader (Filter-Max F5, Molecular Devices, San Jose, CA, USA). All samples were tested in triplicate and the average value of three blanks (no Abs) was subtracted from the reads. The cut-off was determined as two times a mean OD value of the blank controls.

Experimental *Plasmodium* infection of birds

Ethics statement

All procedures were performed at the Nature Research Centre in Vilnius, Lithuania, according to Lithuanian and International Guiding Principles for Biomedical Research Involving Animals (2012). Infection experiments and other procedures were reviewed and approved by the Lithuanian State Food and Veterinary Service, Ref. No. 2020/07/24-G2-84 and International Research Cooperation Agreement between the Biological Station “Rybachy” and the Nature Research Centre (25/05/2010, 04/09/2015). The assessment of the animal health and all described procedures were implemented by trained professionals (under licenses 2012/02/06-No-208, and 2016/01.29-No-344).

Experimental Plasmodium infection of birds

For quantification of anti-α-Gal Abs in sera samples (see below), domestic canaries (*Serinus canaria domestica*) were experimentally infected with *P. relictum* (genetic lineage SGS1) (n = 3 canaries) and *P. homocircumflexum* (COLL4) (n = 5 canaries) following a protocol described by Palinauskas et al. (16). Blood samples used for sera extraction and Abs

quantification were collected from *P. relictum*-infected (At 0, 14, 22 and 38, days post infection) and *P. homocircumflexum*-infected (At 0, 16, 24 and 36, days post infection) birds.
For quantification of α-Gal levels on different *Plasmodium* species (see below), Eurasian siskins (*Carduelis spinus*) were experimentally infected with *P. ashfordi* (GRW2), *P. relictum* (SGS1) and *P. homocircumflexum* (COLL4) following a protocol described by Palinauskas et al. (16). Blood samples used for parasite protein extraction were collected from *P. ashfordi* -infected (At 330 day post infection), *P. relictum*-infected (At 16 day post infection), and *P. homocircumflexum*-infected (At 12 day post infection) birds. One infected blood sample per *Plasmodium* species was used for α-Gal quantification.
Each experimental bird was sub-inoculated with a mixture (0.10 mL) of infected blood, 3.7% sodium citrate and 0.9% saline in proportion 4:1:5 into the pectoral muscles. Each bird from the different groups received approximately $1x10^5$ mature meronts of each *Plasmodium* species. Microscopic examination was used to quantify parasitemia every 4 days in smears of blood collected from the brachial vein.

Blood samples and microscopic examination

Blood samples were taken from birds by puncturing brachial vein and using microcapillaries. A small drop of blood was used to make smears for microscopy to estimate the development of parasites in the blood. Smears were air-dried, fixed with absolute methanol and stained as described in (17). A fraction of blood (20-30 μL) was placed in SET-buffer for molecular analysis (PCR) to confirm the lineage in recipient birds.
An Olympus BX61 light microscope (Olympus, Japan) was used to examine the blood smears. Parasitemia was calculated as a percentage by actual counting of the number of parasites per 10.000 erythrocytes as described by Godfrey et al. (18).

Determination of anti-α-Gal Abs in sera samples

Sera extraction

Blood samples (100 μL) were used to obtain serum for immunological analysis. Before centrifugation the blood was incubated for 2 h at RT, allowing it to coagulate. Then samples were centrifuged at 5000xG for 5 min and serum separated in microtube and kept in a freezer at -15C until processing.

Indirect ELISA for relative quantification anti-α-Gal IgY levels in canary sera

Levels of anti-Galα1-3Gal and anti-Galα1-3Galβ1-4GlcNAc IgY in canary sera were measured as previously described (15), with some modifications. 96-wells ELISA plates (Thermo Scientific, Waltham, MA, USA) were coated with 100 µL/well of either Galα1-3Gal or Galα1-3Galβ1-4GlcNAc linked to HSA (Dextra Laboratories, Reading, UK) at a concentration of 0.5 µg/mL in buffer carbonate/bicarbonate (0.5 M, pH = 9.6) and incubated overnight at 4 °C. Wells were washed three times with 100 µL /well of PBST and then blocked with 100 µL of 1% HSA/PBS for 2 h at RT. After three washes, serum samples diluted in PBS (1:50) were added and incubated for 1 h at 37 °C and 150 rpm. Plates were washed three times and HRP-conjugated Abs goat anti-turkey IgY (Mybiosource, San Diego, CA, USA) diluted at 1:1000 in 0.5 % HSA/PBS (100 µL/well) were added and incubated for 1 h at RT and 150 rpm. Plates were washed three times and the reaction was developed by adding 100 µL ready-to-use TMB solution (Promega, Madison, WI, USA) at RT for 20 min and 150 rpm in the dark, and then stopped with 50 µL of 0.5 M H_2SO_4. The OD was measured at 450 nm using an ELISA plate reader (Filter-Max F5, Molecular Devices, San Jose, CA, USA). All samples were tested in duplicate and the average value of two blanks (no Abs) was subtracted from the reads. The cut-off was determined as two times a mean OD value of the blank controls.

Quantification of α-Gal levels on different *Plasmodium* species

Protein extraction and quantification

For protein extraction, blood samples from birds infected *P. ashfordi*, *P. relictum* and *P. homocircumflexum* were homogenized in 300 µl PBS with 1% Triton X-100 (Sigma-Aldrich) using steel balls and the homogenizer Precellys 24 Dual (Bertin, France) at 6000 rpm for 30s (× 3) each. Total proteins extracted from *Sus scrofa* kidney samples and α-Gal linked to Bovine Serum Albumin (α-Gal-BSA) were used as positive controls. Total proteins extracted from a kidney sample from α-Gal-deficient (α-Gal KO) *S. scrofa* were used as negative control. Proteins were quantified using the bicinchoninic acid assay (BCA) (Thermo Fisher, Massachusetts, United States) with BSA as standard.

Quantification of α-Gal levels on different* Plasmodium *species

α-Gal levels were quantified using an inhibition ELISA as previously reported by Lu et al. (19) with modifications as in Lima-Barbero et al. (20). Briefly, increasing concentrations (5, 10, and 20 ng/μl) of total proteins were incubated 2h at RT and ovbernight 4°C with the anti-α-Gal monoclonal Ab (mAb) M86 (Enzo Life Sciences, Farmingdale, NY, USA). The relative amount of unbound mAb M86 was then measured by ELISA using α-Gal-BSA (Dextra Laboratories, Reading, United Kingdom) as coating antigen (200 ng/well). The goat anti-mouse IgM-HRP (Sigma-Aldrich, Missouri, United States) was used as secondary Ab and the O.D. was measured at 450 nm with a Multiskan FC ELISA reader (Thermo Fisher, Massachusetts, United States). Three replicates were used for each sample. The average value of the blanks was subtracted from all reads before further analysis. The percentage inhibition (%) was calculated as ([O.D. mAb M86 alone] − [O.D. mAb M86 incubated with X ng/μl proteins]) × 100 / [O.D. mAb M86 alone], where X corresponds to the various concentrations of total protein extracts used for incubation with mAb M86 in the ELISA.

Acknowledgements

We thank Angelika Schnieke and Konrad Fischer (Livestock Biotechnology, School of Life Sciences Weihenstephan, Technische Universität München, Freising, Germany) for providing kidney samples from α-Gal-deficient (α-Gal KO) *S. scrofa*.

References

1. Kohl KD, Amaya J, Passement CA, Dearing MD, McCue MD. Unique and shared responses of the gut microbiota to prolonged fasting: a comparative study across five classes of vertebrate hosts. FEMS. *Microbiol Ecol*. (2014) 90:883-894. doi: 10.1111/1574-6941.1244.

2. Dewar ML, Arnould JPY, Krause L, Dann P, Smith SC. Interspecific variations in the faecal microbiota of Procellariiform seabirds. *FEMS Microbiol Ecol*. (2014) 89:47-55. doi: 10.1111/1574-6941.12332.

3. Kreisinger J, Čížková D, Kropáčková L, Albrecht T. Cloacal Microbiome Structure in a Long-Distance Migratory Bird Assessed Using Deep 16sRNA Pyrosequencing. *PLoS One* (2015) 10:e0137401. doi: 10.1371/journal.pone.0137401.

4. Koskey AM, Fisher JC, Traudt MF, Newton RJ, McLellan SL. Analysis of the gull fecal microbial community reveals the dominance of Catellicoccus marimammalium in relation to culturable Enterococci. *Appl Environ Microbiol* (2014) 80:757-765. doi: 10.1128/AEM.02414-13.

5. Roggenbuck M, Bærholm Schnell, I, Blom N, Bælum J, Bertelsen MF, et al. The microbiome of New World vultures. *Nat Commun* (2014) 5:5498. doi: 10.1038/ncomms6498

6. Xu P, Liu P, Zhou C, Shi Y, Wu Q, et al. A Multi-Omics Study of Chicken Infected by Nephropathogenic Infectious Bronchitis Virus. *Viruses* (2019) 11:1070. doi: 10.3390/v11111070. PMID.

7. Adhikari B, Jun SR, Kwon YM, Kiess AS, Adhikari P. Effects of Housing Types on Cecal Microbiota of Two Different Strains of Laying Hens During the Late Production Phase. *Front Vet Sci* (2020) 7:331. doi: 10.3389/fvets.2020.00331.

8. Leinonen R, Sugawara H, Shumway M. The sequence read archive. *Nucleic Acids Res* (2011) 39, 2010–2012. doi: 10.1093/nar/gkq1019.

9. Bolyen E, Dillon M, Bokulich N, Abnet, CC, Al-Ghalith GA, et al. Reproducible, interactive, scalable, and extensible microbiome data science using QIIME 2. *Nat Biotechnol* (2019) 37, 852–857. doi: 10.1038/s41587-019-0209-9.

10. Callahan BJ, McMurdie PJ, Rosen MJ, Han AW, Johnson AJ, et al. DADA2: High-resolution sample inference from Illumina amplicon data. *Nat Methods* (2016) 13, 581–583. doi: 10.1038/nmeth.3869.

11. Bokulich NA, Kaehler BD, Rideout JR, Dillon M, Bolyen, E, et al. Optimizing taxonomic classification of marker-gene amplicon sequences with QIIME 2’s q2-feature-classifier plugin. *Microbiome* (2018) 6, 90. doi: 10.1186/s40168-018-0470-z.

12. Yarza P, Yilmaz P, Pruesse E, Glöckner FO, Ludwig W, et al. Uniting the classification of cultured and uncultured bacteria and archaea using 16S rRNA gene sequences. *Nat Rev Microbiol* (2014) 12, 635-645. doi: 10.1038/nrmicro3330.

13. Douglas GM, Maffei VJ, Zaneveld J. Yurgel NS, Brown JR, et al. PICRUSt2: An improved and extensible approach for metagenome inference. *BioRxiv* (2019) 672295. doi.org/10.1101/672295.

14. Kanehisa M, Goto S. KEGG: Kyoto encyclopedia of genes and genomes. *Nucleic Acids Res* (2000) 28, 27–30. doi: 10.1093/nar/28.1.27.

15. Mateos-Hernández L, Risco-Castillo V, Torres-Maravilla E, Bermúdez-Humarán LG, Alberdi P, et al. Gut Microbiota Abrogates Anti-α-Gal IgA Response in Lungs and Protects against Experimental Aspergillus Infection in Poultry. *Vaccines (Basel)* (2020) 8:285. doi: 10.3390/vaccines8020285.

16. Palinauskas V, Valkiūnas G, Bolshakov CV, Bensch S. *Plasmodium relictum* (lineage P-SGS1): effects on experimentally infected passerine birds. *Exp Parasitol* (2008) 120, 372–380. doi: 10.1016/j.exppara.2008.09.001.

17. Valkiūnas G, Iezhova TA, Križanauskienė A, Palinauskas V, Sehgal RNM, Bensch S. A comparative analysis of microscopy and PCR-based detection methods for blood parasites. *J Parasitol* (2008) 94:1395–1401. doi:10.1645/GE-1570.1

18. Godfrey RD, Fedynich AM, Pence DB. Quantification of hematozoa in blood smears. *J Wildl Dis* (1987) 23:558–565. doi:10.7589/0090-3558-23.4.558

19. Lu Y, Shao A, Shan Y, Zhao H, Leiguo M, et al. A standardized quantitative method for detecting remnant alpha-Gal antigen in animal tissues or animal tissue-derived biomaterials and its application. *Sci Rep* (2018) 8, 15424. doi: 10.1038/s41598-018-32959-1.

20. Lima-Barbero JF, Sánchez MS, Cabezas-Cruz A, Mateos-Hernández L, Contreras M. et al. Clinical gamasoidosis and antibody response in two patients infested with *Ornithonyssus bursa* (Acari: Gamasida: Macronyssidae). *Exp Appl Acarol* (2019) 78:555-564. doi: 10.1007/s10493-019-00408-x.
